# Physical Activity as a Mediator of the Relationship Between Mediterranean Diet Adherence and Anxiety Symptoms in Chilean Adolescents: A Cross-Sectional Study

**DOI:** 10.3390/children13060825

**Published:** 2026-06-17

**Authors:** Felipe Caamaño-Navarrete, Claudio Hernández-Mosqueira, Guido Contreras-Diaz, Indya del-Cuerpo, Daniel Jerez-Mayorga, Tomás Herrera-Valenzuela, Eduardo Guzmán-Muñoz, Jordan Hernandez-Martinez, Pablo Valdés-Badilla, Cristian Núñez-Espinosa, Pedro Delgado-Floody

**Affiliations:** 1 Physical Education Career, Universidad Autónoma de Chile, Temuco 4780000, Chile; felipe.caamano@uautonoma.cl; 2 Departamento de Ciencias de la Educación, Universidad del Bio-Bio, Chillan 3780000, Chile; chernandez@ubiobio.cl; 3 Escuela de Kinesiología, Facultad de Ciencias de la Rehabilitación y Calidad de Vida, Universidad San Sebastián, Lago Panguipulli 1390, Puerto Montt 5501842, Chile; guido.contreras@uss.cl; 4 Department of Physical Education and Sports, Faculty of Sport Sciences, Sport and Health University Research Institute (iMUDS), University of Granada, 18071 Granada, Spain; delcuerpo@ugr.es (I.d.-C.); djerezmayorga@ugr.es (D.J.-M.); 5 Exercise and Rehabilitation Sciences Institute, Faculty of Rehabilitation Sciences, Universidad Andres Bello, Santiago 7591538, Chile; 6 Department of Physical Activity, Sports and Health Sciences, Faculty of Medical Sciences, Universidad de Santiago de Chile (USACH), Santiago 8370003, Chile; tomas.herrera@usach.cl; 7 School of Kinesiology, Faculty of Health, Universidad Santo Tomás, Talca 3460000, Chile; eguzmanm@santotomas.cl; 8 Department of Physical Activity Sciences, Universidad de Los Lagos, Osorno 5290000, Chile; jordan.hernandez@ulagos.cl; 9 Department of Education, Faculty of Humanities, Universidad de la Serena, La Serena 1700000, Chile; 10 Department of Physical Activity Sciences, Faculty of Education Sciences, Universidad Católica del Maule, Talca 3460000, Chile; pvaldes@ucm.cl; 11 Sports Coach Career, Faculty of Life Sciences, Universidad Viña del Mar, Viña del Mar 2520000, Chile; 12 Escuela de Medicina, Universidad de Magallanes, Punta Arenas 6200000, Chile; cristian.nunez@umag.cl; 13 Centro Asistencial Docente y de Investigación, Universidad de Magallanes, Punta Arenas 6200000, Chile; 14 Red Interuniversitaria de Envejecimiento Saludable de Latinoamérica y Caribe (RIES-LAC), Punta Arenas 6200000, Chile; 15 Department of Physical Education, Sport and Recreation, Universidad de La Frontera, Temuco 4811230, Chile

**Keywords:** mediterranean diet, physical activity, adolescents, anxiety, depression, mental health, mediation analysis

## Abstract

**Highlights:**

**What are the main findings?**

- Greater adherence to the Mediterranean diet is directly linked to lower levels of depression, stress, and overall psychological distress in Chilean adolescents.
- Physical activity acts as a significant partial mediator in the relationship between Mediterranean diet adherence and anxiety symptoms.

**What are the implications of the main findings?**

- The association between diet quality and anxiety is shared with physical activity levels, indicating that these two lifestyle factors interact specifically to modulate anxiety.
- Clinical and school-based interventions should implement combined lifestyle prescriptions targeting both nutritional habits and physical activity to effectively address adolescent psychological distress.

**Abstract:**

Background: Adolescent mental health is a global concern, with lifestyle factors such as diet and physical activity (PA) playing a crucial role. While the Mediterranean Diet adherence (MDA) is known for its neuroprotective benefits on mental health, the mechanisms by which they are related remain unclear. Therefore, the aim of this study was twofold: (1) to examine the associations between MDA, PA, and screen time (ST) with anxiety symptoms, depression, stress, and total psychological distress in Chilean adolescents; and (2) to determine whether PA mediates the relationship between MDA and anxiety symptoms. Methods: A cross-sectional study was conducted with 322 Chilean school-aged adolescents 158 males and 164 females (14.98 ± 1.96 years). Mental health outcomes (depression, anxiety, and stress) and lifestyle behaviors, including MDA, PA, and ST, were comprehensively assessed using validated self-reported questionnaires. Results: After adjusting for age and sex, multiple linear regression models showed that higher MDA was significantly and inversely associated with anxiety (b = −0.23, *p* = 0.044), stress (b = −0.25, *p* = 0.022), and total psychological distress (b = −0.72, *p* = 0.022). Conversely, ST was identified as a consistent risk factor, positively predicting higher levels of anxiety (b = 0.45, *p* = 0.008), stress (b = 0.42, *p* = 0.008), and total distress (b = 1.11, *p* = 0.014). Furthermore, PA was inversely linked to anxiety (b = −0.35, *p* = 0.013) and successfully mediated the relationship between MDA and anxiety symptoms (Indirect Effect = −0.047, 95% CI: −0.10 to −0.01). No significant mediation effects were observed for depression or stress. Conclusions: The present study identifies robust inverse associations between MDA and symptoms of anxiety, depression, and stress in Chilean adolescents. A key finding is the specific pattern of relationships observed: while higher MDA is directly linked to lower levels of depression and stress, its association with anxiety is shared with levels of PA. Despite these findings, the cross-sectional nature of the study limits the establishment of causal relationships, and further longitudinal research is needed to confirm these directional pathways.

## 1. Introduction

Mental health is a cornerstone of the human experience. According to the World Health Organization (WHO), it is far more than the absence of illness; it is a state of well-being where individuals can realize their potential, navigate daily stressors, work productively, and contribute meaningfully to their communities [1]. Mental health disorders, particularly anxiety, depression, and stress, have become a leading cause of global disease burden [2,3], affecting quality of life and socioeconomic productivity [4]. Complementary to the above, data indicates that the prevalence of mental health problems among young people affects approximately 10 to 20 percent of children and adolescents worldwide [5]. Moreover, a recent study reported that from 1990 to 2021, the standardized global prevalence of Mental health disorders increased by 6.8%, while anxiety disorders exhibited the highest prevalence [6]. Mental health components could have negative consequences on cognitive, emotional, and social development in youth [7]. In this sense, mental problems scores as an adolescent were more likely to be a consistent smoker, have depression and have more self-reported physician-diagnosed mental health conditions as an adult [8].

Recent evidence suggests that lifestyle factors, including dietary patterns and physical activity (PA), play a pivotal role in the modulation of psychological well-being [9,10], through complex biological pathways, such as the regulation of systemic inflammation and the gut–brain axis [10]. In addition, a healthy lifestyle includes PA and a healthy diet pattern, moreover it has been indicated that some definitions also include smoking cessation, social and psychological well-being, and moderate to no alcohol use [11]. The scientific evidence has indicated that an unhealthy lifestyle (i.e., Lifestyle-related factors characterized by poor eating habits and low levels of physical activity,) and high screen time (ST) [12] could increase the likelihood of death from all causes; however, several studies have broadened the view, generating strong evidence that an unhealthy lifestyle is negatively related to poor mental health [13].

Likewise, the evidence has shown that the promotion of PA in schools is fundamental for improving mental health elements [14]. Similarly, another study highlighted that rates of PA decline throughout the school stage, which can have a negative impact on mental health [15]. This aligns with prior research highlighting the crucial role of PA and a healthy lifestyle in mental health benefits [16].

Among dietary patterns, the Mediterranean Diet adherence (MDA), characterized by a high intake of plant-based foods, healthy fats, and antioxidants, has emerged as a robust protective factor against mental distress [3]. A study carried out by Delgado et al. [17] claimed that high diet quality (i.e., MDA) showed an association with better mental health in schoolchildren. Hence, MDA could be a protective factor for mental health in schoolchildren populations [18]. Likewise, scientific evidence reports that better dietary patterns are inversely linked to having depressive symptoms [19]. Indeed, good food habits such as a teenage breakfast and overall diet quality have been associated with better mental health dimensions [20]. Therefore, diet quality is relevant because the literature shows that MDA is associated with reduced psychiatric symptoms and lower risk of mental disorders in paediatric populations [21].

Concurrently, regular PA is recognized for its anxiolytic and antidepressant effects [22], primarily mediated by the release of neurotrophic factors and endorphins [23]. However, most research has examined these lifestyle components in isolation. While it is established that individuals with better dietary habits tend to be more physically active [24], the underlying mechanisms of how these behaviors interact to influence specific mental health dimensions remain poorly understood. Specifically, it is unclear whether the benefits of a healthy diet on mental health are mediated or if they are partially explained by an increase in PA levels, a mechanism known as statistical mediation.

Understanding these association pathways is crucial for clinical practice, as it allows for the development of more targeted interventions [25]. For instance, if PA mediates the relationship between MDA and anxiety but not depression, clinical guidelines should prioritize combined “lifestyle prescriptions” differently depending on the patient’s primary [26]. Despite the known benefits of MDA on physical health, its specific impact on adolescent mental health through lifestyle mediators remains insufficiently explored. Therefore, the aim of this study was twofold: (1) to examine the associations between MDA, PA, and ST with anxiety symptoms, depression, stress, and total psychological distress in Chilean adolescents; and (2) to determine whether PA mediates the relationship between MDA and anxiety symptoms. We hypothesized that: (1) higher MDA and PA would be inversely associated with anxiety, stress, depression and total distress, whereas higher ST would be positively associated with these psychological outcomes; and (2) PA would significantly and partially mediate the relationship between MDA and adolescent anxiety symptoms.

## 2. Materials and Methods

### 2.1. Study Design and Participants

A cross-sectional study was conducted among adolescents from two urban educational centers in the Araucanía Region, Chile. A non-probabilistic convenience sampling strategy was employed based on institutional access and fieldwork feasibility. This region is historically characterized by high socioeconomic vulnerability indices and significant public health priorities, which makes the evaluation of its youth highly relevant for local and regional health strategies.

The minimum statistical sample size was determined through an a priori power analysis using GPower software (version 3.1.9.7) [27]. The calculation was based on an F-test for multiple linear regression (R^2^), assuming a conservative small-to-medium effect size (f^2^ = 0.10), a significance level of 0.05, and a high statistical power (1 − beta) of 0.95. For a model incorporating 5 main predictors (MDA, PA, ST, age, and gender), the analysis indicated that a minimum of 198 participants was required to detect robust associations. Consequently, a final sample of 322 adolescents was targeted and achieved, ensuring adequate statistical power for both the multivariate regression and the subsequent mediation models.

The inclusion criteria for this study were rigorous and designed to ensure the integrity of the research. These criteria included: (i) being a student, to clearly define the target population, (ii) being within the eligible age range of 13 to 18 years old, which captures middle and late adolescent stages and (iii) having provided written informed assent by the student along with the corresponding signed informed consent from their parents or legal guardians prior to the assessments. Similarly, the exclusion criteria were detailed to ensure the suitability of participants for the assessments. These included: (i) any medical contraindication that could impair normal performance in the assessments, thus ensuring that the results accurately reflected the capabilities of the student population under study, and (ii) absence during the assessments or failure to provide informed consent, thereby maintaining the validity and ethical standards of data collection.

This study strictly adhered to the ethical principles outlined in the Declaration of Helsinki (2013), ensuring the protection of participants’ rights in scientific research. Additionally, it received approval from the Ethics Committee of Universidad Autónoma de Chile, Chile (ACTA; No. CEC 13-25), affirming the study’s methodological validity and ethical integrity. Student participation was contingent upon obtaining their signed consent, along with the informed consent of their respective parents or guardians.

### 2.2. Procedures

Data collection was conducted during school hours through self-reported digital questionnaires. Prior to administration, participants were informed about the study’s objectives, the anonymity of their responses, and their right to withdraw at any time. The evaluations were completed individually under the supervision of trained researchers who were present to address any questions or concerns. Specifically, during morning sessions, the mental health and lifestyle assessments were carried out in the schools’ computer laboratories. The instruments were digitally administered via the Google Forms platform, utilizing a specific link that contained all necessary instructions alongside the data collection measures designed for this research.

### 2.3. Instruments

#### 2.3.1. Mental Health Assessment (DASS-21)

Psychological distress symptoms were quantified using the Depression, Anxiety, and Stress Scale (DASS-21). This validated, self-administered questionnaire features a three-dimensional structure specifically engineered to identify and score the severity of affective states related to anxiety, depression, and stress in participants [28]. This three-dimensional self-report scale was designed to evaluate the presence and intensity of emotional states or symptoms of depression, anxiety and stress [29,30,31]. Structurally, the instrument comprises 21 items equally distributed among three independent subscales. The depression scale investigates symptoms such as dysphoria, anhedonia, life devaluation, and a lack of interest; the anxiety dimension screens for physiological arousal, along with general and situational anxiety experiences; and the stress subscale measures irritability, tension, relaxation difficulties, and persistent overthinking. Each item is evaluated using a 4-point frequency Likert scale ranging from 0 (not applying to the respondent) to 3 (applying heavily or most of the time) based on the participant’s experiences over the preceding week. Cumulative subscale scores span from 0 to 21 points, where lower values signify lesser symptom severity. Clinical cutoff thresholds were established at >5 points to detect depression and stress manifestations, and >4 points for identifying anxiety symptoms [32]. This instrument has the advantages of being a self-report scale, brief, easy to administer and answer, and easy to interpret. These questionnaires have been used with Chilean students [33] and presented adequate psychometric properties [34]. DASS-21 has shown good internal consistency (Cronbach’s alpha > 0.80) in adolescent subjects [35]. To interpret symptom severity using the DASS-21, participants’ cumulative scores were categorized into distinct clinical tiers. Depressive symptoms were classified as mild (5–6 points), moderate (7–10 points), severe (11–13 points), or extremely severe (14 points or higher). For the anxiety dimension, the thresholds were defined as mild for a score of 4, moderate for 5–7, severe for 8–9, and extremely severe for any outcome reaching 10 or more points. Finally, stress manifestations were stratified into mild (8–9 points), moderate (10–12 points), severe (13–16 points), and extremely severe (17 points or more) presentation levels. The cut-off points correspond to those indicated in previous research [36,37].

#### 2.3.2. Mediterranean Diet Adherence (MDA)

To evaluate dietary patterns and establish nutritional quality linked to MDA, the validated Krece Plus questionnaire was implemented [38]. This screening tool serves as an efficient method to identify potential nutritional imbalances and behavioral risks associated with childhood and adolescence (e.g., questions regarding daily consumption of fruits, vegetables, or commercially baked goods). The instrument is structurally composed of 15 dichotomous items with ‘yes’ or ‘no’ response options, where each answer is assigned a value of +1 or −1 depending on established dietary guidelines. Based on the cumulative scoring system, participant outcomes are stratified into three distinct tiers of dietary quality: optimal food habits are represented by scores between 8 and 12, moderate habits requiring adjustment are indicated by scores from 4 to 7, and poor dietary patterns are defined by a range of 0 to 3 points [38]. The Krece Plus instrument has been previously validated and applied to students [39].

#### 2.3.3. Lifestyle

Screen time and physical activity

To evaluate PA and sedentary patterns (i.e., ST), the Krece Plus PA test was administered individually to the adolescents under researcher supervision [40]. This brief instrument categorizes overall lifestyle habits by examining weekly after-school PA alongside daily screen time (ST) dedicated to television or video games. Methodologically, ST values were reverse-coded to align the cumulative score with a healthier lifestyle profile. Consequently, ST tracking ranging from less than an hour to four or more hours daily was scored from 5 down to 1 point, respectively, whereas PA frequencies were scored directly. The aggregated points establish three lifestyle categories stratified by sex: an optimal or good lifestyle (≥9 points for males, ≥8 points for females), a regular lifestyle (6–8 points for males, 5–7 points for females), and an unfavorable or bad lifestyle (≤5 points for males, ≤4 points for females).

### 2.4. Statistical Analysis

All the statistical analyses were performed with SPSS statistical software version 23.0 (SPSS^TM^ Inc., Chicago, IL, USA). The alpha level was set at *p* < 0.05 for statistical significance. First, the normality of the data distribution was evaluated using the Kolmogorov–Smirnov test. All primary variables (Anxiety symptoms, MDA score, and PA levels) showed a normal distribution (*p* > 0.05). Descriptive statistics (mean and standard deviation) were calculated for all variables. Second, independent samples *t*-tests were conducted to explore potential sex differences in mental health and lifestyle variables. Third, Pearson’s correlation was chosen to first identify relationship between the main variables (Anxiety symptoms, MDA score, and PA levels) before proceeding to the mediation analysis.

Fourth, to evaluate the association between lifestyle factors and multiple mental health outcomes (treated as continuous variables: Anxiety, Depression, Stress, and Total DASS score), multiple linear regression models were performed. To ensure the validity of the multiple linear regression models, multicollinearity among the independent variables was evaluated using the Variance Inflation Factor (VIF) and tolerance values. In these multivariate models, independent variables (MDA score, screen time, and PA levels) were entered as continuous variables, while Model 2 was further adjusted for age (continuous variable) and sex (coded as a dummy variable: 0 = Male, 1 = Female) as potential confounders. For these models, unstandardized coefficients (b), 95% confidence intervals (95% CI), and *p*-values were reported. To evaluate the explanatory power of the proposed models, we calculated the coefficient of determination (R^2^). Additionally, Cohen’s f^2^ was calculated as a measure of effect size for all multiple linear regression models to determine the practical significance of the observed associations, which is calculated as f^2^ = R^2^/(1 − R). According to Cohen’s guidelines, f^2^ values of 0.02, 0.15, and 0.35 were utilized to define small, medium, and large effect sizes, respectively. To ensure the reliability of the multivariate models, multicollinearity was assessed using the Variance Inflation Factor (VIF) and tolerance values.

To test the hypotheses, a mediation analysis was conducted using the macro/interface process v. 3.3 for SPSS v. 23 and the bootstrapping method with a resampling rate of 5000 [35]. Dietary habits (i.e., MDA) were entered as the independent variable (X), PA as the mediator (M), and Mental Health dimensions (Anxiety, Depression, Stress, and DASS Total) as the dependent variables (Y). Statistical significance for indirect effects was determined using a non-parametric bootstrapping procedure. A 95% confidence interval (CI) that did not include zero was considered evidence of a significant mediation effect. Additionally, the proportion of the total effect explained by the mediator was calculated using the formula:

(ab/c)*100,

where ab represents the indirect effect and c represents the total effect. All models were adjusted for sex and age as covariates [41].

## 3. Results

### 3.1. Sample Characteristics

The descriptive analysis and sex comparisons are summarized in Table 1. The participants had a mean age of 14.98 ± 1.96 years, ranging from 13 to 18 years. Regarding sex distribution, the sample was nearly balanced, consisting of 158 males (49%) and 164 females (51%). No significant differences were found in age and MDA between sexes (*p* > 0.05). However, females exhibited significantly higher scores across all mental health dimensions, including total mental health (*p* = 0.004), anxiety (*p* < 0.001), depression (*p* = 0.038), and stress (*p* = 0.032). Regarding lifestyle habits, males reported significantly higher levels of PA (4.18 vs. 3.16 h/week; *p* < 0.001) and more hours of ST (3.33 vs. 2.87 h/day; *p* = 0.024) compared to their female counterparts.

### 3.2. Correlation Analysis

The correlation (Table 2) revealed a significant relation between lifestyle factors and mental health. MDA showed a significant and positive correlation with PA levels (r = 0.19) and was inversely related to all mental health dimensions: total mental health (r = −0.18), anxiety (r = −0.18), depression (r = −0.16), and stress (r = −0.16). Notably, PA was specifically and more strongly correlated with anxiety (r = −0.20) compared to depression and stress, where the relations were not statistically significant. Regarding ST, no significant correlations were found with mental health scores or other lifestyle habits in this sample (*p* > 0.05).

### 3.3. Multiple Linear Regression Analysis

Prior to the multivariate analysis, multicollinearity diagnostics were verified. For all independent variables (MDA, screen time, and PA), the Variance Inflation Factor (VIF) values ranged from 1.008 to 1.043, while tolerance values remained above 0.95. These diagnostics confirm the absence of multicollinearity, indicating that the independent variables are not redundant and that the model’s coefficients are stable. The results of the multiple linear regression analysis are presented in Table 3. In the crude models (Model 1), MDA, PA, and ST were significantly associated with anxiety, stress, and total mental health. When the models were adjusted for age and sex (Model 2), the association of MDA remained statistically significant for anxiety (b = −0.23, *p* = 0.046), stress (−0.25, *p =* 0.022), and total Mental health (b = −0.72, *p* = 0.022). Regarding sedentary behavior, ST emerged as a consistent risk factor, significantly predicting higher levels of anxiety (b = 0.45, *p* = 0.008), stress (b = 0.42, *p* = 0.008), and total mental health (b = 1.11, *p =* 0.014). Notably, PA was linked to anxiety (b = −0.35, *p* = 0.013), but its association with depression and stress did not reach statistical significance in the adjusted models.

The explanatory power of the models was assessed using the coefficient of determination (R^2^) and Cohen’s f^2^ for effect size. For Anxiety, the adjusted model explained 10.9% of the variance (R^2^ = 0.109, *p* = 0.024) with a small-to-medium effect size (f^2^ = 0.12). Stress and Total Mental Health adjusted models explained 7.5% (R^2^ = 0.075, f^2^ = 0.08) and 8.5% (R^2^ = 0.085, f^2^ = 0.09) of the variance, respectively. Finally, the Depression model showed the lowest explanatory power (R^2^ = 0.042, f^2^ = 0.04).

**Figure 1:**
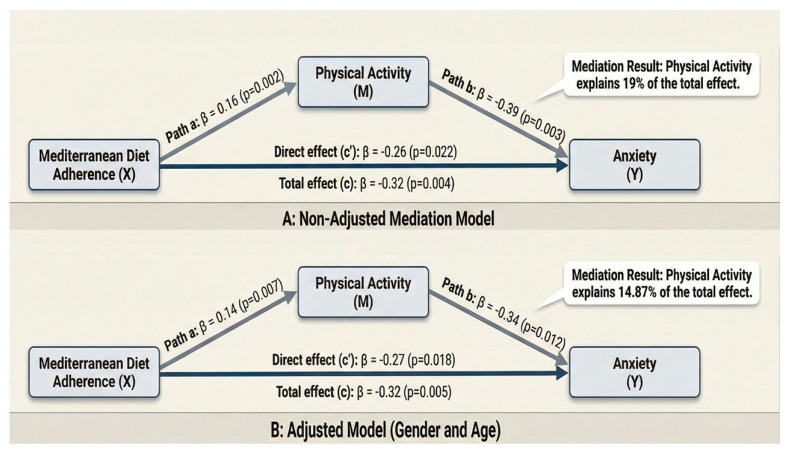
Mediation analysis of physical activity in the relationship between Mediterranean diet adherence and anxiety symptoms in Chilean adolescents, showing non-adjusted (**A**) and adjusted (**B**) models.

To explore whether PA mediates the relationship between MDA and Anxiety levels, a mediation analysis was conducted (Table 4). The mediation model results indicated that MDA was linked to PA (b = 0.16, SE = 0.05, *p* = 0.002). Furthermore, both MDA (b = −0.26, SE = 0.11, *p* = 0.022) and PA (b = −0.39, SE = 0.13, *p* = 0.003) were negatively and significantly associated with Anxiety levels. The total effect of MDA on Anxiety was significant (c = −0.32, *p* = 0.004, 95% CI; −0.54 to −0.10). Upon including PA as a mediator in the model, the direct effect of Dietary Habits on Anxiety remained statistically significant (c` = −0.26, *p* = 0.022, 95% CI; −0.48 to −0.04), indicating the presence of a partial mediation. Finally, the analysis of indirect effects confirmed that PA significantly mediated the relationship between MDA and anxiety (IE = −0.06, SE = 0.03, 95% CI; −0.12 to −0.02). Specifically, this indirect pathway accounted for 19% of the total effect (Figure 1A). Adjusting for sex and age as potential confounders. The results indicated that MDA was positively linked to PA (b = 0.14, SE = 0.05, *p* = 0.007). Furthermore, both MDA (b = −0.27, SE = 0.11, *p* = 0.018) and PA (b = −0.34, SE = 0.13, *p* = 0.012) were negatively and significantly associated with Anxiety levels. The total effect of MDA on Anxiety was significant (c = −0.32, 95% CI: −0.54, −0.10, *p* = 0.005). Upon including PA as a mediator, the direct effect of MDA on Anxiety remained statistically significant (c` = −0.27, *p* = 0.018, 95% CI: −0.49 to −0.05), indicating a partial mediation. Finally, the analysis of indirect effects confirmed that PA significantly mediated the relationship between MDA and Anxiety (IE = −0.047, SE = 0.025, 95% CI: −0.10 to −0.01), accounting for 14.87% of the total effect. Notably, the significance of this mediation model remained robust after controlling for the influence of sex and age (Figure 1B).

In Table 5, mediation analyses were also conducted to evaluate whether PA mediated the impact of MDA on Depression, Stress, and Total mental health. Although MDA was significantly linked to PA in all models (a = 0.158, *p* = 0.002), PA did not emerge as a significant predictor for Depression (*p* = 0.303), Stress (*p* = 0.306), or overall DASS scores (*p* = 0.065) when controlling for MDA. Consequently, the bootstrapping procedure confirmed the absence of significant indirect effects for Depression (IE = −0.022; 95% CI; −0.074 to 0.019), Stress (IE = −0.019; 95% CI; −0.07 to 0.02), and Total mental health (IE = −0.10; 95% CI; −0.25 to 0.00).

## 4. Discussion

The aim of this study was twofold: (1) to examine the associations between MDA, PA, and ST with anxiety symptoms, depression, stress, and total psychological distress in Chilean adolescents; and (2) to determine whether PA mediates the relationship between MDA and anxiety symptoms. Our findings provide novel insights by demonstrating that while a high MDA has a robust association across all psychological domains (anxiety, depression, and stress), the underlying mechanisms of action differ significantly. Specifically, PA emerged as a significant mediator only for the relationship between MDA and anxiety.

These results reinforce previous evidence on the differential role of lifestyle factors in adolescent mental health, particularly highlighting the positive role of diet quality. In fact, even after adjusting for age and sex, MDA maintained a consistent association with anxiety, stress, and overall mental health. This finding suggests that the beneficial relationship between MDA and mental health does not rely exclusively on other healthy behaviors, such as engagement in PA, but rather stems from intrinsic neurobiological mechanisms linked to its nutritional composition [42]. In this regard, nutrients characteristic of the MDA, such as omega-3 fatty acids, B vitamins, polyphenols, and other antioxidant compounds, have been shown to modulate greater emotional regulation and resilience to stress [43]. In this regard, evidence has highlighted that adherence to MDA has been positively related to mental health variables in the school context [42]. Likewise, growing evidence has related both the onset and symptoms of different mental health problems to lifestyle factors such as diet quality; however, further research is needed to rigorously establish causal inferences between diet quality and mental health, and not just to establish cross-sectional relationships [44]. In addition, higher adherence to this dietary pattern has been associated with a lower risk of developing depressive and anxious symptoms, even after controlling for sociodemographic and lifestyle variables [18].

Our most notable finding was the significant link between MDA and anxiety symptoms through PA levels. This partial mediation suggests that adolescents with healthier dietary habits are more likely to engage in regular PA, which in turn contributes to lower anxiety symptoms by reducing autonomic arousal and the somatic manifestations of stress [45]. Complementary to the above, a systematic literature review found a positive association between better MDA and lower levels of anxiety [46]. Another study reported that better MDA was associated with better levels of mental health in the school setting, which may be relevant for considering food quality as a relevant variable in the education system [47]. From a physiological standpoint, this relationship may be explained by the synergistic action between key components of the MDA and the neurobiological effects of PA [48]. Likewise, a healthy lifestyle characterized by MDA plus PA can be a key element to consider due to the positive relationship they may have with mental health [49].

The finding that PA functioned as a significant mediator only in the anxiety model offers an important perspective on the specificity of psychophysiological mechanisms underlying different emotional domains. This pattern suggests that the physiological regulation derived from PA, particularly its role on some relevant physiological variables, appears to be especially effective in mitigating anxiety-related symptoms, which are primarily characterized by heightened physiological arousal [50]. Also, the evidence has been compelling in indicating that higher levels of affect may be associated with better levels of mental health in schoolchildren [14]. In addition, physical exercise-induced modulation of components of anxiety, depression and stress [51].

Conversely, PA did not significantly mediate the association between MDA and depression or stress. This finding underscores the role of the MDA pattern as a key factor directly associated with better mental health outcomes. Moreover, accumulating scientific evidence has shown that adherence to Mediterranean dietary patterns is consistently linked to various dimensions of psychological well-being in adolescents [52]. In this regard, previous research has indicated a positive association between better scores on the MDA and mental health [16,19]. Our findings are consistent with another study conducted on schoolchildren, which reported that better eating habits were associated with better levels of mental health [53]. In this sense, it has been indicated that nutrients characteristic of this dietary model have been shown to enhance serotonergic synthesis, reduce neuroinflammation, and promote neuroplasticity, thereby it has been related to affective and cognitive stress regulation independent of physical exertion [54].

Consistent with global epidemiological trends, female adolescents in our sample exhibited significantly higher scores across all mental health dimensions, including overall psychological distress, anxiety, depression, and stress. This pattern aligns with prior research demonstrating that females are more vulnerable to internalizing symptoms during adolescence developmental period, marked by hormonal, cognitive, and social transitions that heighten emotional reactivity [55]. Biologically, fluctuations in estrogen and progesterone interact with serotonergic and dopaminergic systems, increasing susceptibility to mood dysregulation and stress sensitivity [56]. Psychosocially, adolescent girls tend to engage in more ruminative coping and interpersonal stress processing, while also facing greater sociocultural pressures related to body image, academic achievement, and social relationships, all of which compound emotional strain [57].

Notably, recent evidence suggests that healthy lifestyle behaviors, particularly adherence to MDA and regular PA, may buffer these vulnerabilities, thereby improving emotional regulation and reducing stress reactivity in female adolescents [58]. However, the persistence of higher distress among females underscores the need for sex-sensitive interventions that integrate nutritional, behavioral, and psychosocial components to promote emotional resilience during adolescence.

These findings highlight the importance of promoting healthy lifestyle behaviors as integral components of adolescent mental health strategies. The favorable impact of MDA, independent of PA, suggests that nutritional interventions should be prioritized alongside physical exercise programs in school and community settings. Encouraging adherence to Mediterranean-style eating patterns and regular PA may foster emotional regulation, mitigate anxiety, and build psychological resilience during this critical developmental stage. Moreover, the observed sex differences underscore the need for sex-sensitive prevention programs that integrate nutritional education, stress management, and emotional coping skills tailored to the specific vulnerabilities of adolescent girls.

### Limitations and Strengths

On the other hand, this study possesses several important strengths that should be highlighted. First, it addresses a critical gap in the literature by examining the joint interaction of dietary patterns and PA on multiple dimensions of mental health within a South American adolescent population, which remains underrepresented compared to European or North American cohorts. Second, rather than relying solely on simple bivariate correlations, this study employs a robust statistical mediation framework (via Hayes’ PROCESS macro). This approach allows for a deeper understanding of the underlying behavioral mechanisms, demonstrating how PA acts as a key pathway through which MDA influences anxiety. Finally, these findings carry practical utility, offering measurable and timely evidence that can guide educational institutions and public health policymakers in designing comprehensive, dual-component lifestyle interventions to safeguard adolescent psychological well-being.

Despite its strengths, this study is not without limitations. Primarily, its cross-sectional design precludes the establishment of definitive causal relationships. Although the mediation model identifies a statistical pathway between MDA, PA, and anxiety symptoms, these results must be interpreted with caution. It remains plausible that alternative directions of association exist; for instance, adolescents with lower anxiety levels or greater emotional well-being may be more naturally predisposed to engage in regular PA.

Furthermore, certain population context variables, such as the urban or rural setting and the objective nutritional status of participants, were not recorded, which may limit the generalizability of the findings to different geographical or physiological contexts.

Fourth, physical activity levels and daily screen time were assessed using the Krece Plus short questionnaire. While this instrument is highly practical, validated, and widely used for large-scale epidemiological school-based research due to its low participant burden, it carries inherent limitations common to subjective, self-reported tools. Exclusive reliance on these methods introduces recall bias and social desirability bias, which may lead adolescents to overreport physical activity or underreport screen time. Crucially, screen time was evaluated in a very general manner based on declared hours, which may not accurately capture multi-device usage or background exposure, potentially oversimplifying actual digital behaviors. Furthermore, the study lacks objective, device-based measurements such as accelerometry or pedometers for PA, which are necessary to provide precise data regarding intensity, duration, and energy expenditure. Consequently, our behavioral findings should be interpreted as general trends rather than exact quantitative measurements, and future research must consider combining subjective questionnaires with objective monitoring technologies. In addition, the omission of several potential confounding variables that were not collected during the initial assessment, such as socioeconomic status, body mass index, nutritional status, sleep quality, family environment, and clinical history (including medication use or previous mental health diagnoses). The absence of these factors restricts the comprehensive interpretation of the regression models. Sixth, the external validity and generalizability of our findings are restricted due to the sampling design. Data collection was limited to two educational centers within a single region in southern Chile (The Araucanía), utilizing a non-random convenience sampling approach. Consequently, these results cannot be directly extrapolated to the broader Chilean adolescent population, nor to youth from different socio-cultural or geographic backgrounds. While these findings offer a valuable exploratory window into regional youth health behaviors, future research should implement stratified, multi-center random sampling methods across diverse socioeconomic and geographical strata to confirm national trends.

Future longitudinal studies are required to confirm the directional flow and the explanatory mechanisms proposed in our statistical model. Additionally, the reliance on self-reported data may introduce recall bias. Furthermore, our model has not considered some variables such as socioeconomic level and body mass index, psychiatric history, medication use, or family environment. This study included the use of a convenience sample, and the results are not necessarily representative of the national population.

## 5. Conclusions

The present study identifies robust inverse associations between MDA and symptoms of anxiety, depression, and stress in Chilean adolescents. A key finding is the specific pattern of relationships observed: while higher MDA is directly linked to lower levels of depression and stress, its association with anxiety is shared with levels of PA. Furthermore, screen time emerged as a consistent risk factor for psychological distress, while the benefits of PA were found to be uniquely significant for managing anxiety. For adolescents presenting with anxiety, a combined intervention focusing on both nutritional quality and increased PA is essential. Conversely, for depression and stress, improving dietary habits remains a critical standalone target. These findings underscore the importance of school-based health programs that integrate balanced nutrition and active lifestyles to safeguard adolescent mental well-being. While a combined prescription of diet and PA is ideal for global mental health, it may be particularly indispensable for participants presenting with high anxiety. For those with depressive symptoms, focusing on nutritional quality appears to be a critical first-line intervention that provides benefits even when PA levels are low.

## Figures and Tables

**Table 1:** Descriptive characteristics of the study participants and differences by sex.

Variables	Total (n = 322)	Male (n = 158)	Female (n = 164)	*p*-Value
Age (years)	14.98 ± 1.96	15.07 ± 1.93	14.90 ± 1.99	0.428
MDA (score)	5.64 ± 2.66	5.79 ± 2.65	5.50 ± 2.66	0.327
Physical Activity (h/week)	3.63 ± 2.23	4.18 ± 2.23	3.16 ± 2.23	<0.001
Screen Time (h/day)	3.09 ± 1.79	3.33 ± 1.79	2.87 ± 1.79	0.024
Mental Health (score)	23.80 ± 13.36	21.61 ± 13.36	25.90 ± 13.36	0.004
Anxiety (score)	7.22 ± 5.03	6.21 ± 5.03	8.20 ± 5.03	<0.001
Depression (score)	7.39 ± 5.11	6.78 ± 5.11	7.98 ± 5.11	0.038
Stress (score)	9.18 ± 4.66	8.61 ± 4.66	9.73 ± 4.66	0.032

Data are presented as Mean ± Standard Deviation. MDA: Mediterranean Diet Adherence. Significant values are considered as *p* < 0.05.

**Table 2:** Bivariate correlations between lifestyle factors and mental health dimensions.

Variables	1	2	3	4	5	6
1. MDA	1					
2. Physical Activity	0.18 **	1				
3. Screen Time	−0.08	−0.14 *	1			
4. Anxiety	−0.18 **	−0.22 **	0.19 **	1		
5. Depression	−0.15 *	−0.13 *	0.11	0.68 **	1	
6. Stress	−0.16 **	−0.15 *	0.17 **	0.72 **	0.75 **	1

Data are presented as Pearson’s correlation coefficients (r). MDA: Mediterranean Diet Adherence. * *p* < 0.05; ** *p* < 0.01.

**Table 3:** Multiple linear regression analysis for mental health dimensions according to Lifestyle.

Independent Variables	Model 1 (Crude)		Model 2 (Adjusted)	
	b (95% CI)	*p*-Value	b (95% CI)	*p*-Value
	Anxiety			
MDA	−0.23 (−0.46, −0.00)	0.046	−0.23 (−0.46, −0.00)	0.044
Screen Time	0.41 (0.07, 0.74)	0.017	0.45 (0.12, 0.78)	0.008
Physical Activity	−0.42 (−0.69, −0.15)	0.002	−0.35 (−0.62, −0.08)	0.013
R^2^	0.082 (8.2%)		0.109 (10.9%)	
	Depression			
MDA	−0.24 (−0.48, −0.00)	0.048	−0.24 (−0.48, 0.05)	0.055
Screen Time	0.20 (−0.14, 0.55)	0.240	0.24 (−0.10, 0.59)	0.173
Physical Activity	−0.17 (−0.45, 0.11)	0.249	−0.11 (−0.40, 0.17)	0.438
R^2^	0.031 (3.1%)		0.042 (4.2%)	
	Stress			
MDA	−0.26 (−0.47, −0.05)	0.018	−0.25 (−0.46, −0.04)	0.022
Screen Time	0.39 (0.07, 0.69)	0.014	0.42 (0.11, 0.73)	0.008
Physical Activity	−0.17 (−0.42, 0.08)	0.194	−0.11 (−0.36, 0.15)	0.398
R^2^	0.059 (5.9%)		0.075 (7.5%)	
	Mental Health			
MDA	−0.73 (−1.34, −0.11)	0.020	−0.72 (−1.33, −0.10)	0.022
Screen Time	1.03 (0.10, 1.89)	0.028	1.11 (0.22, 2.00)	0.014
Physical Activity	−0.75 (−1.47, −0.02)	0.042	−0.57 (−1.33, 0.16)	0.128
R^2^	0.064 (6.4%)		0.085 (8.5%)	

Data shown represent unstandardized beta (b) and 95% Confident Interval (95% CI): Model 1, non-adjusted (crude); Model 2, adjusted by Age and Sex. MDA: Mediterranean Diet Adherence. Significant values are considered *p* < 0.05.

**Table 4:** Mediation analysis of Physical Activity in the relationship between Mediterranean Diet Adherence and Anxiety, adjusted for Sex and Age.

	Path	(b)	SE	t	*p*-Value	95% CI
Direct Effects						
MDA -> Physical Activity	a	0.16	0.05	3.58	0.002	[0.06, 0.26]
Adjusted	a	0.14	0.05	2.71	0.007	[0.04, 0.24]
Physical Activity -> Anxiety	b	−0.39	0.13	−3.34	0.003	[−0.64, −0.14]
Adjusted	b	−0.34	0.13	−2.53	0.012	[−0.61, −0.08]
MDA -> Anxiety (Direct effect)	c`	−0.26	0.11	−2.81	0.022	[−0.48, −0.04]
Adjusted	c`	−0.27	0.11	−2.39	0.018	[−0.49, −0.05]
Total Effect						
MDA -> Anxiety (Total effect)	c	−0.32	0.112	−2.81	0.004	[−0.54, −0.10]
Adjusted	c	−0.32	0.112	−2.81	0.005	[−0.40, −0.10]
Covariates						
Gender (Female)		1.48	0.61	2.43	0.016	[0.28, 2.68]
Age		−0.13	0.16	−0.85	0.394	[−0.44, 0.17]
Indirect Effect		Effect	SE	95% CI		Result
MDA -> PA -> Anxiety	a*b	−0.06	0.03	[−0.12, −0.02]		Partial Mediation
Adjusted	a*b	−0.05	0.03	[−0.10, −0.01]		Partial Mediation

b: unstandardized coefficient; SE: standard error; CI: confidence interval; MDA: Mediterranean Diet Adherence; PA: Physical Activity. Significant values are considered as *p* < 0.05.

**Table 5:** Mediation analysis results of Physical Activity on the relationship between Mediterranean Diet Adherence and Mental Health dimensions.

Outcome (Y)	Total Effect (c)	Direct Effect (c′)	Indirect Effect	95% CI	Result
			(a*b)		
Depression	−0.25 *	−0.23	−0.022	[−0.07, 0.02]	No Mediation
Stress	−0.30 **	−0.28 **	−0.019	[−0.06, 0.02]	No Mediation
Mental health Total	−0.87 **	−0.76 *	−0.103	[−0.25, 0.00]	No Mediation

Note: All models were adjusted for Sex and Age. Coefficients (β) are unstandardized. MDA: Mediterranean Diet Adherence; PA: Physical Activity. 95% confidence intervals (95% CI) were calculated using 5000 samples. * *p* < 0.05; ** *p* < 0.01.

## Data Availability

The data presented in this study are available on request from the corresponding author (P.D.-F.). The data are not publicly available due to privacy and ethical restrictions involving school-aged participants, as per the protocols approved by the Ethics Committee of Universidad Autónoma de Chile (ACTA; No. CEC 13-25).
